# Biopharmaceutical Study on Nobiletin-Loaded Amorphous Solid Dispersion with Improved Hypouricemic Effect

**DOI:** 10.3390/molecules26154447

**Published:** 2021-07-23

**Authors:** Takuya Nihei, Eri Ushiro, Hideyuki Sato, Satomi Onoue

**Affiliations:** Laboratory of Biopharmacy, School of Pharmaceutical Sciences, University of Shizuoka, 52-1 Yada, Suruga-ku, Shizuoka 422-8526, Japan; ggle.t.n@gmail.com (T.N.); m12017@u-shizuoka-ken.ac.jp (E.U.); h.sato@u-shizuoka-ken.ac.jp (H.S.)

**Keywords:** amorphous solid dispersion, dissolution, hyperuricemia, nobiletin, oral absorption

## Abstract

The present study aimed to develop an amorphous solid dispersion of nobiletin (ASD/NOB) using hydroxypropyl cellulose-SSL (HPC-SSL) to improve the pharmacokinetic properties and hypouricemic effect of NOB. ASD/NOB was prepared by the freeze-drying method (ASD/NOB). ASD/NOB was characterized with a focus on crystallinity, dissolution, pharmacokinetic behavior, and hypouricemic action in a rat model of hyperuricemia. ASD/NOB showed significant improvement in dissolution behavior, as evidenced by a 4.4-fold higher dissolved NOB concentration than crystalline NOB at 2 h in distilled water. After the oral administration of ASD/NOB (50 mg NOB/kg) in rats, higher systemic exposure to NOB was observed with an 18-fold enhancement in oral bioavailability, and the *T*_max_ value of orally administered ASD/NOB was 60% shorter than that of orally administered crystalline NOB. In a rat model of hyperuricemia, orally dosed ASD/NOB showed an improved hypouricemic effect by a 16% reduction in the plasma uric acid level compared with orally administered crystalline NOB. Based on these findings, ASD/NOB may be an efficacious dosage option to improve the nutraceutical potential of NOB for the treatment of hyperuricemia.

## 1. Introduction

Hyperuricemia is a pathological condition caused by the overproduction and/or underexcretion of uric acid, the final product in the purine catabolic pathway [1]. Long-standing hyperuricemia is often associated with several severe diseases [2], and treatment of hyperuricemia has attracted much interest by virtue of preventing those complications. Inhibitors of xanthine oxidase (XO), a purine catabolism enzyme, are one of the effective therapeutic approaches for the treatment of hyperuricemia [3]. However, these therapeutic agents also often induce side effects, leading to a reduced quality of life [4]. Recently, there has been growing interest in nobiletin (NOB), a natural compound derived from citrus plants (genus *Citrus*), in terms of its attractive biological activities, including anti-inflammatory effects and anti-XO action [5]. Thus, NOB might be a promising nutraceutical agent for the treatment of hyperuricemia. However, NOB exhibits low oral bioavailability (BA) due to poor water solubility (16.2 μg/mL in water at 37 °C), probably resulting in insufficient clinical outcomes [6,7].

With the aim of improving the therapeutic potential of NOB, a very limited number of studies have been conducted to design formulations of NOB, including a self-microemulsifying system, self-assembling proliposomes, and solid dispersion (SD) [8,9]. In particular, the SD approach is one of the simplest manufacturing techniques and could enhance dissolution properties and BA by reducing the particle size and improving the wettability of compounds with poor water solubility [10,11,12]. On the basis of the molecular arrangement, the SD technique can be classified as either a one-phase or two-phase system [13]. The one-phase and two-phase systems are defined as a solid solution and a solid dispersion mainly prepared by freeze-drying and wet-milling, respectively. Previously, our group developed an amorphous SD formulation (ASD) of NOB prepared by wet-milling technology (wmASD/NOB) [6,7]. In terms of the dissolution profile, cyclosporine A (an active pharmaceutical ingredient with immunosuppressive effects) prepared by freeze-drying is superior to the wet-milled SD formulation because the cyclosporine A molecules are emitted easily when hydrophilic polymers are dissolved in water [14], and this prompted us to develop ASD/NOB using freeze-drying technology (ASD/NOB). Although the strategic application of ASD for NOB prepared by the freeze-drying method might result in enhanced hypouricemic action of NOB at a lower dose, little is known regarding its feasibility.

The present study was undertaken to clarify the nutraceutical potential of ASD/NOB as an anti-hyperuricemic agent. The physicochemical properties were characterized for ASD/NOB with a focus on morphology, crystallinity, and dissolution behavior. The pharmacokinetic profile of NOB was assessed after the oral administration of ASD/NOB to rats. The hypouricemic effects of crystalline NOB and ASD/NOB were evaluated on the basis of plasma uric acid levels in a rat model of hyperuricemia.

## 2. Results and Discussion

### 2.1. Appearance and Crystallinity of NOB Samples

**Figure 1:**
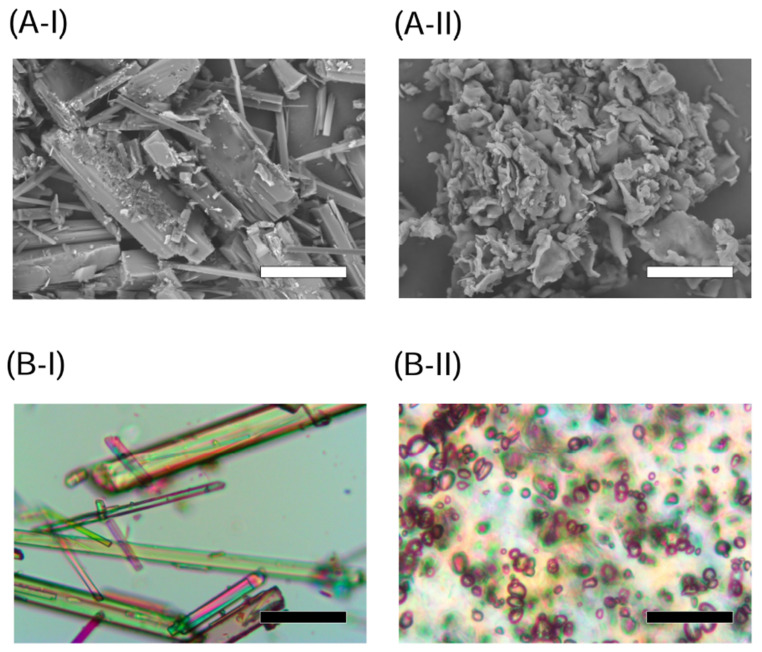
Morphological observations of NOB samples. (**A**) Scanning electron microscopy and (**B**) polarized light microscopy of (I) crystalline NOB and (II) ASD/NOB. White and black bars represent 50 μm.

**Figure 2:**
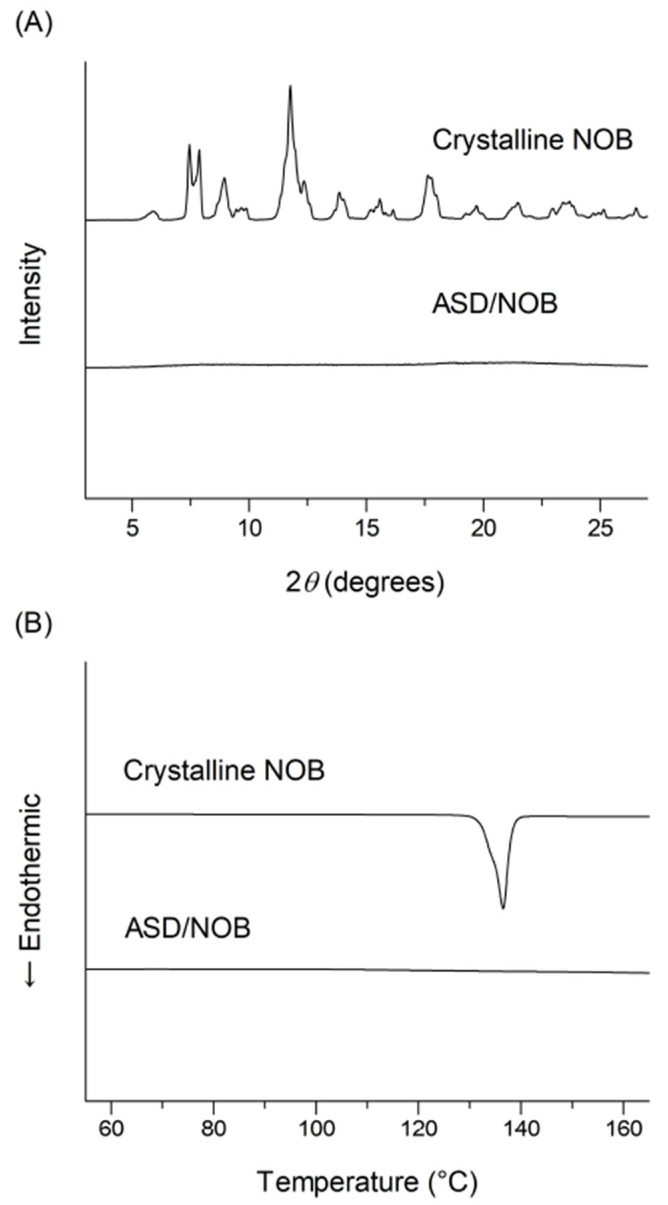
Physicochemical characterization of NOB samples. (**A**) Powder X-ray diffraction and (**B**) differential scanning calorimetry of crystalline NOB and ASD/NOB.

Chemicals in an amorphous state tend to be unstable during long-term storage, and SD techniques are often used to prepare stable amorphous formulations [15]. A previous study demonstrated that an HPC-SSL-based ASD formulation with 50 wt% NOB prepared by freeze-drying resulted in high storage stability without recrystallization and transition in the morphology after storage for 4 weeks under accelerated conditions (40 °C/75% RH) [6]. Thus, ASD/NOB was prepared with the same ratio of crystalline NOB and HPC-SSL to achieve both amorphization and high stability under the accelerated conditions. According to SEM images of crystalline NOB and ASD/NOB (Figure 1(A-I,A-II)), crystalline NOB showed relatively large needle-like particles, while needle-shaped NOB powder was negligible in ASD/NOB, suggesting that micronized NOB was dispersed into the polymer in ASD/NOB after lyophilization. PLM micrographs of these samples demonstrated the high crystallinity of NOB as evidenced by intense birefringence (Figure 1(B-I)), whereas little birefringence was observed in ASD/NOB (Figure 1(B-II)). The crystallinity of NOB samples was further characterized by PXRD and DSC. Crystalline NOB revealed several sharp intense peaks in the PXRD pattern (Figure 2A). By contrast, there was a typical halo diffraction pattern for ASD/NOB. DSC thermograms of crystalline NOB showed a thermal event at 137 °C (Figure 2B). However, no thermal event was observed in DSC thermograms of ASD/NOB within the examined temperature range. From these findings, NOB in ASD/NOB was found to be in an amorphous state, and amorphization of NOB resulted in better wettability. Amorphized NOB with a high wettability could disperse quickly in dissolution media, possibly leading to enhancing dissolution properties of NOB. 

### 2.2. Dissolution Testing of NOB Samples

**Figure 3:**
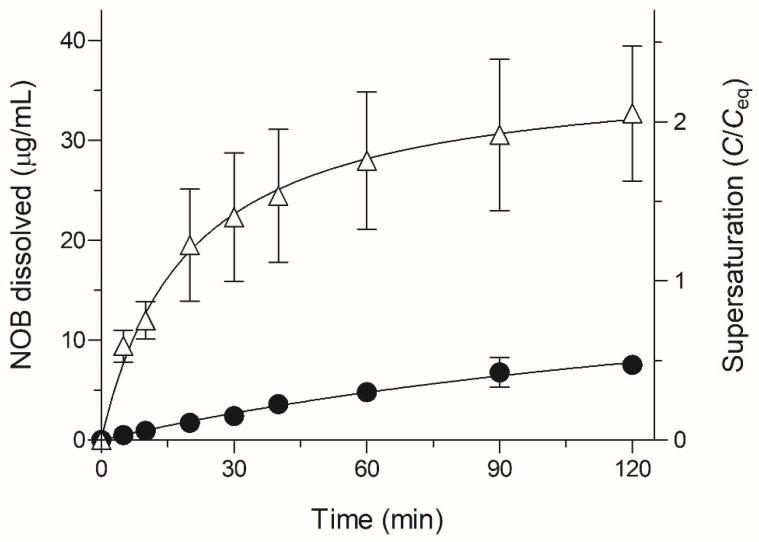
Dissolution profiles of NOB samples in distilled water. ●, Crystalline NOB; △, ASD/NOB. Each bar represents the mean ± SD of three independent experiments.

Theoretically, NOB with a non-electrolyte chemical structure has the physicochemical properties of pH-independent solubility. Water was selected as a dissolution medium because the nature of water is similar to gastrointestinal fluid regarding the buffer capacity, an important factor of drug dissolution [16]. Dissolution testing in water for NOB samples could be accessed appropriately for evaluating the differences of in vivo dissolution between crystalline NOB and ASD/NOB. To clarify the possible improvement in dissolution behavior of NOB in ASD/NOB, dissolution testing was performed in water for up to 2 h (Figure 3). Crystalline NOB showed poor dissolution behavior, as evidenced by the dissolved NOB concentration of 7.5 μg/mL (46% of the solubility in water) even after 2 h. However, there was a significant improvement in the dissolution behavior of ASD/NOB as evidenced by the dissolved NOB concentration of 33 μg/mL, achieving a twofold higher concentration of the dissolved NOB than the equilibrium solubility (C_eq_, 16.2 μg/mL in water at 37 °C) of NOB at 2 h. Higher variation in the concentration of the dissolved NOB was observed in ASD/NOB than in crystalline NOB. Precipitation of NOB could be caused for the supersaturation of NOB at an invisible level, probably leading to high variation in the dissolution of NOB in ASD/NOB. In a previous study, drug release of a physical mixture of crystalline NOB and HPC-SSL was similar to that of crystalline NOB after 2 h in distilled water, suggesting that HPC-SSL might not act as a potent solubilizing agent for NOB [6]. In general, HPC-SSL increases the dissolution rate of poorly water-soluble drugs because of the enhanced wettability of the formulation, and the ASD approach for NOB using HPC-SSL as a polymer could enhance the saturated solubility and dissolution of NOB. According to the Mark–Houwink–Sakurada equation, viscosity is proportional to molecular weight, and drug dispersion is inversely proportional to the viscosity of an inert polymer according to the Stokes–Einstein equation [17,18]. Therefore, the lower the viscosity of the molecules, the higher the dispersion they exhibit in aqueous media. The use of HPC-SSL with low molecular weight (15,000–30,000 Da) and viscosity (2.0–2.9 mPa.s) resulted in the enhanced dissolution of NOB in ASD/NOB compared with crystalline NOB. Moreover, wmASD/NOB (NOB formulations prepared by wet-milling technique in previous work) and ASD/NOB exhibited 3.6- and 4.4-fold greater concentrations of dissolved NOB than crystalline NOB, respectively. Dissolution studies were carried out for wmASD/NOB and ASD/NOB under different experimental conditions in terms of the dissolution test apparatus, water volume, and agitation force in the water, so the dissolution behavior of these NOB formulations may not be able to be compared directly. However, regardless of the preparation methods for the NOB formulations, amorphized NOB in ASD formulations reached similar levels after 2 h and finally became saturated, suggesting that the freeze-drying technique for NOB might also result in significant improvement in its pharmacokinetic behavior. From these observations, HPC-SSL-based ASD formulation with 50 wt% of NOB exhibited high storage stability for 4 weeks under the condition of 40 °C/75% RH and enhanced the dissolution properties of NOB. The optimal content ratio of NOB and HPC-SSL remains unclear, and further studies are required on the optimization of their ratio for improved storage stability and dissolution properties of NOB.

### 2.3. Pharmacokinetic Study of NOB Samples

**Figure 4:**
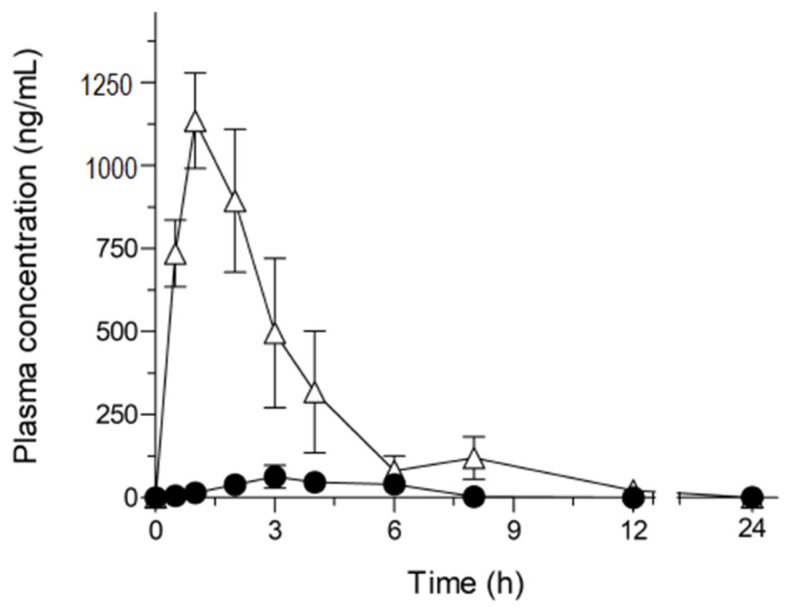
Plasma concentration-time profiles of NOB after oral administration of NOB samples (50 mg NOB/kg) in rats. ●, Crystalline NOB; △, ASD/NOB. Data represent the mean ± SE of six experiments.

The observations of dissolution properties of ASD/NOB prompted us to investigate the possible improvement in oral absorption, so a pharmacokinetic study in rats was carried out for orally dosed crystalline NOB and ASD/NOB (50 mg NOB/kg) (Figure 4 and Table 1). As reported previously, there was slight systemic exposure to NOB after the oral administration of crystalline NOB. On the basis of the AUC_0–24_ value (1.2 μg.h/mL) of intravenously administered NOB (0.5 mg/mL) in our previous study [7], the BA of crystalline NOB in rats was calculated to be 0.20%. On the other hand, orally-dosed ASD/NOB led to a marked improvement in *C*_max_ and AUC_0–24_ values of NOB, leading to an 18-fold enhancement in the absolute BA of NOB with the use of ASD technology. The oral administration of ASD/NOB also resulted in the shortening of *T*_max_ by 1.8 h compared with crystalline NOB. The rapid oral absorption might facilitate the rapid onset of the nutraceutical function of NOB in clinical use, possibly leading to better clinical outcomes. In a previous study, dissolution testing was carried out for ASD formulations with several loading amounts of tranilast, a therapeutic agent for inflammatory diseases, in the range of 40–70% *w*/*w*. While a drug concentration of under 50% exhibited rapid dissolution behavior, a poor dissolution behavior was observed in the ASD formulation with a loading amount of tranilast over 60% in a drug load-dependent manner, suggesting that there is an optimal ratio of a drug and a polymer in terms of dissolution and pharmacokinetic properties [19]. In the present study, ASD/NOB with 50 wt% of NOB exhibited significant improvement in the pharmacokinetic behavior of NOB, which was caused by the sufficiently high supersaturation of NOB observed in the dissolution testing. However, further optimization in the content ratio of NOB and HPC-SSL would be needed for enhanced dissolution and pharmacokinetic behaviors of NOB.

In previous studies, orally dosed NOB formulations showed improved pharmacokinetic behavior in rats compared with crystalline NOB, resulting in 7- and 13-fold enhancements of absolute BA at doses of 2 and 20 mg NOB/kg, respectively [6,7]. Orally administered ASD/NOB led to an 18-fold improvement in BA of NOB at a dose of 50 mg NOB/kg. NOB is known to inhibit *P*-glycoprotein (*P*-gp), a 170 kDa glycoprotein that is expressed in plasma membranes, and functions to exclude drugs from cells, leading to a decrease in the intracellular concentration [20]. Generally, *P*-gp substrate drugs often cause *P*-gp saturation with high systemic exposure. The present study, taken together with previous observations, demonstrated that enhanced systemic exposure to NOB induced the saturation of *P*-gp-mediated exclusion, possibly resulting in increased BA of NOB in comparison with that of NOB formulations in previous studies. Based on these findings, improved pharmacokinetic behavior of NOB might lead to enhanced nutraceutical function, particularly regarding the hypouricemic effects of NOB.

### 2.4. Hypouricemic Effects of NOB Samples in Rats

**Figure 5:**
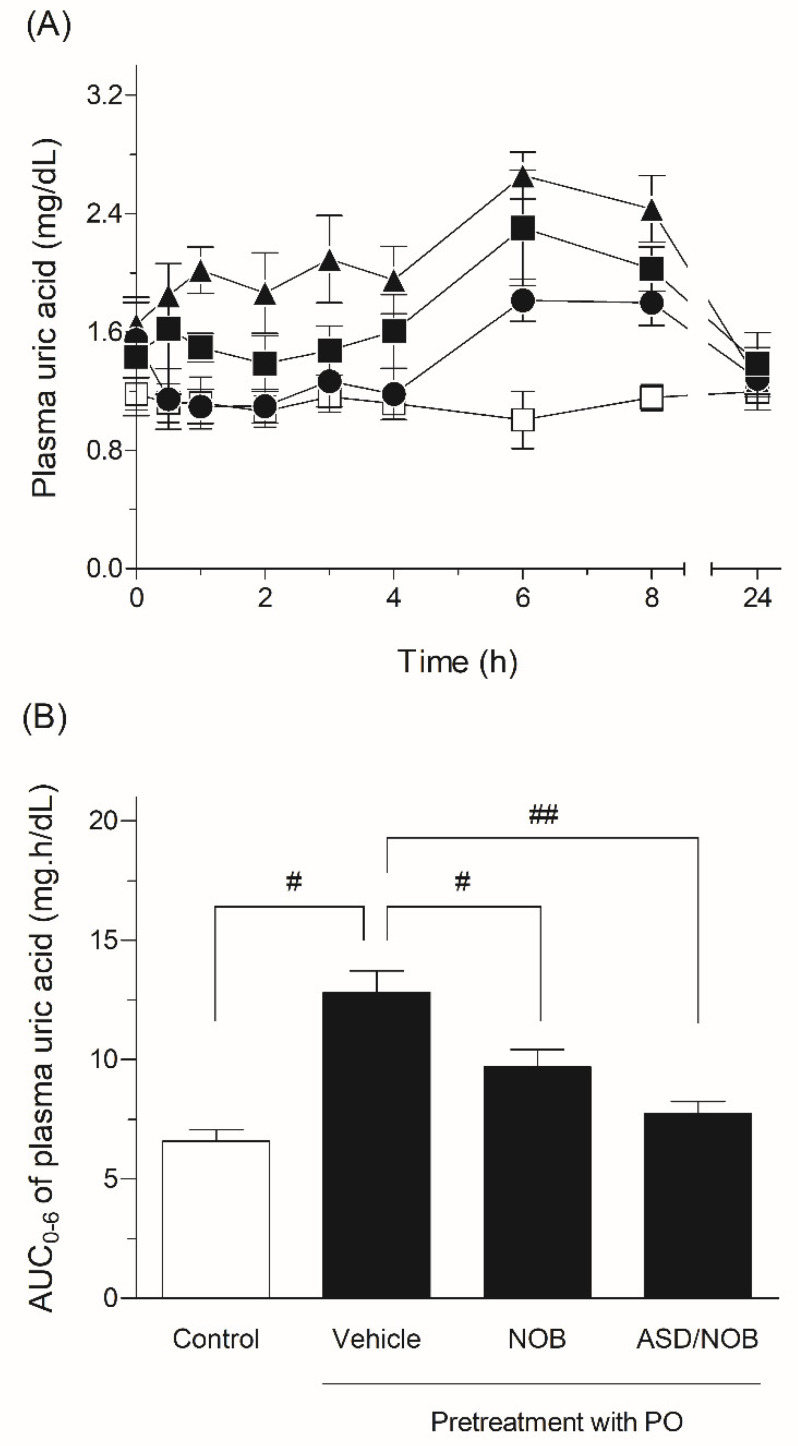
Pharmacodynamic behavior in a rat model of hyperuricemia induced by PO. (**A**) Plasma uric acid level-time profiles in rats after the oral administration of NOB samples. □, Control; ▲, PO (250 mg/kg, i.p.); ■, PO with orally-dosed crystalline NOB (50 mg/kg); ●, PO with orally administered ASD/NOB (50 mg NOB/kg). (**B**) AUC_0–6_ values of plasma uric acid in rats with orally administered NOB samples. ^#^, *p* ≤ 0.05 and ^##^, *p* ≤ 0.01 between indicated loops. Data represent the mean ± SE of six experiments.

There are various studies on the nutraceutical effects of NOB such as antioxidant, anti-inflammatory, antibacterial, and hepatoprotective effects [21,22,23]. NOB is expected to act as an anti-hyperuricemic agent through the modulation of inflammatory and oxidative events. Although previous studies demonstrated that NOB exhibited inhibitory activity on XO in in vitro assays, little is known about the hypouricemic effect of NOB in in vivo experiments [5]. The attractive nutraceutical effect of NOB could be concentration-dependent in the plasma, and comparative experiments were conducted to assess anti-hyperuricemic activity after the oral administration of crystalline NOB or ASD/NOB (Figure 5). In the present study, the hypouricemic effects of NOB samples were assessed in a rat model of hyperuricemia induced by PO. Uric acid is converted into allantoin by uricase and finally excreted through urea in most mammals [24]. Uricase could be inhibited by PO, a selective uricase inhibitor, and PO has been employed to prepare a rat model of hyperuricemia. After PO challenge, the plasma uric acid level showed a significant increase, suggesting a state of hyperuricemia in rats. By contrast, the oral administration of NOB samples significantly decreased the plasma uric acid level in a rat model of hyperuricemia, and orally dosed ASD/NOB exhibited a higher pharmacological effect than orally administered crystalline NOB, as evidenced by 24 and 40% reductions in plasma uric acid levels with crystalline NOB and ASD/NOB at doses of 50 mg/kg, respectively. These observations were in agreement with the improved pharmacokinetic behavior of ASD/NOB, leading to enhanced delivery of NOB at the site of XO, especially in gastrointestinal and liver sites [25]. Considering the inhibitory activity of NOB on XO (*IC*_50_: 108 μM) in an in vitro experiment, the hypouricemic action of NOB in rats was higher than expected. The reasons for the higher nutraceutical function of NOB than our expectation remain unclear, and the active metabolites of NOB might be attributed to the inhibitory activity on XO, possibly resulting in the hypouricemic action of orally administered NOB in rats. From these observations, improved dissolution and pharmacokinetic behaviors of NOB could result in an enhanced distribution to gastrointestinal and liver sites and thereby the enhanced hypouricemic effect of NOB. 

## 3. Material and Methods

### 3.1. Chemicals

Nobiletin (NOB) was kindly provided by the National Agriculture and Food Research Organization (Shizuoka, Japan). Hydroxypropyl cellulose-SSL (HPC-SSL) (15,000–30,000 Da) was purchased from FUJIFILM Wako Pure Chemical Industries (Osaka, Japan). All other chemicals were purchased from commercial sources.

### 3.2. Preparation for ASD Formulation of NOB

NOB (100 mg) and HPC-SSL (100 mg) were dissolved in 1,4-dioxane solution (20 mL), and the solution was mixed well and frozen at −80 °C. The sample was freeze-dried using an FD-81 freeze dryer (Tokyo Rikakikai, Tokyo, Japan).

### 3.3. NOB Determination

The measurement method for NOB was described in previous work [6,7]. Briefly, the concentration of NOB was determined by ultra-performance liquid chromatography with electrospray ionization mass spectrometry (UPLC/ESI-MS) analysis (Waters, Milford, MA, USA). Waters Acquity UPLC BEH C18 (particle size: 1.7 μm, column size: 2.1 × 50 mm; Waters) was used. Samples were separated using a gradient mobile phase consisting of Milli-Q containing 5 mM ammonium acetate and acetonitrile, and analysis was carried out using selected ion recording (SIR), depending on mass-to-charge ratio of NOB and dexamethasone (internal standard). 

### 3.4. Microscopic Experiments

Representative scanning electron microscopic images of NOB samples were taken with a scanning electron microscope, Miniscope^®^ TM 3030 (Hitachi, Tokyo, Japan), and representative polarized light microscopic images of NOB samples were filmed with a CX41 microscope (Olympus Co., Ltd., Tokyo, Japan), as reported previously [6,7].

### 3.5. Crystallinity

Powder X-ray diffraction patterns were collected using a Mini Flex II (Rigaku Corporation, Tokyo, Japan), and differential scanning calorimetry was performed using a DSC Q1000 (TA Instruments, New Castle, DE, USA), as reported in our previous studies [6,7].

### 3.6. Dissolution Test

A dissolution test was carried out for 2 h in 50 mL of distilled water with constant stirring of 50 rpm using a magnetic stirrer SST-66 (Shimadzu, Kyoto, Japan) at 37 °C. Each NOB sample (4.0 mg of NOB) was weighed in the dissolution vessel. The collected samples were centrifuged at 15,000× *g* for 5 min, and the supernatants were diluted with a 2-fold volume of acetonitrile immediately after centrifugation. 

### 3.7. Pharmacokinetic Studies

#### 3.7.1. Animals and Drug Administration

Male Sprague–Dawley rats (Japan SLC, Shizuoka, Japan) weighing 180 ± 20 g were housed three per cage in the laboratory with free access to food and water. The animal experiments were conducted according to the guidelines approved by the Institutional Animal Care and Ethical Committee of the University of Shizuoka (Approval No. 156145).

#### 3.7.2. Plasma Concentration of NOB

Blood samples were taken in a volume of 500 μL from the tail vein in the indicated periods after oral administration of the NOB samples. The pretreatment methods and measurements for the plasma concentration of NOB were described in previous work [6,7].

### 3.8. Pharmacodynamic Studies

#### 3.8.1. Rat Model of Hyperuricemia

An animal model of hyperuricemia induced by potassium oxonate (PO), a uricase inhibitor, was used to evaluate the hypouricemic effect of NOB in rats according to the procedure of a previous report with some modification [26]. Briefly, PO (250 mg/kg) was intraperitoneally administered to rats 1 h before the oral administration of NOB samples and was then administered to rats two more times at 2 h intervals. 

#### 3.8.2. Measurement of Uric Acid

Uric acid levels in plasma samples were measured using a uric acid assay kit (Wako, Osaka, Japan). Samples were incubated for 5 min at 37 °C, and absorption at 555 nm (main wavelength) and 700 nm (subwavelength) was measured using SAFIRE (TECAN, Männedorf, Switzerland).

### 3.9. Statistical Analysis

For statistical comparisons, one-way analysis of variance (ANOVA) followed by Dunnett’s multiple comparison test was used. A *p*-value of less than 0.05 was considered significant for all analyses.

## 4. Conclusions

In the present study, ASD/NOB exhibited rapid dissolution behavior in comparison with crystalline NOB. Improved biopharmaceutical properties of NOB were observed for ASD/NOB, resulting in enhanced hypouricemic effects of NOB in a rat model of hyperuricemia. Based on these findings, the ASD formulation approach might be an attractive dosage option for NOB to improve its nutraceutical potential for the treatment of hyperuricemia.

## Figures and Tables

**Table 1:** Pharmacokinetic parameters of orally dosed NOB samples in rats.

	*C*_max_ (μg/mL)	*T*_max_ (h)	AUC_0–24_ (μg·h/mL)	BA (%)
Crystalline NOB (50 mg/kg)	0.087 ± 0.029	3.0 ± 0.58	0.23 ± 0.086	0.20 ± 0.072
ASD/NOB (50 mg-NOB/kg)	1.2 ± 0.16	1.2 ± 0.15	4.1 ± 0.80	3.5 ± 0.67

*C*_max_, maximum concentration; *T*_max_, time to maximum concentration; AUC_0–24_, area under the curve of blood concentration vs. time from *t* = 0 to *t* = 24; BA, oral bioavailability. Data represent the mean ± SE of six experiments.

## Sample Availability

Samples of the compounds are not available.

## Abbreviations

ASD: amorphous solid dispersion; AUC_0–24_, area under the curve of blood concentration vs. time from *t* = 0 to *t* = 24 after oral administration; C_eq_, equilibrium solubility; *C*_max_, maximum concentration; DSC, differential scanning calorimetry; HPC, hydroxypropyl cellulose; *P*-gp, *P*-glycoprotein; PLM, polarized light microscopy; PO, potassium oxonate; PXRD, powder X-ray diffraction; RH, relative humidity; SEM, scanning electron microscopy; *T*_max_, time to maximum concentration; and UPLC/ESI-MS, ultra-performance liquid chromatography equipped with electrospray ionization mass spectrometry.
